# Experiences of integrating community volunteers as extensions of the primary care team to help support older adults at home: a qualitative study

**DOI:** 10.1186/s12875-020-01165-2

**Published:** 2020-05-16

**Authors:** Jessica Gaber, Doug Oliver, Ruta Valaitis, Laura Cleghorn, Larkin Lamarche, Ernie Avilla, Fiona Parascandalo, David Price, Lisa Dolovich

**Affiliations:** 1 Department of Family Medicine, McMaster University, David Braley Health Sciences Centre, 1280 Main Street West, Hamilton, ON L8S 4K1 Canada; 2 School of Nursing, McMaster University, 1280 Main Street West, Hamilton, ON L8S 4K1 Canada; 3 Department of Medicine, McMaster University, 1280 Main Street West, Hamilton, ON L8S 4K1 Canada

**Keywords:** Volunteers, Primary care, Older adults, Home visits, Qualitative research, Interprofessional teams

## Abstract

**Background:**

Increasing the integration of community volunteers into primary health care delivery has the potential to improve person-focused, coordinated care, yet the use of volunteers in primary care is largely unexplored. Health Teams Advancing Patient Experience: Strengthening Quality (Health TAPESTRY) is a multi-component intervention involving trained community volunteers functioning as extensions of primary care teams, supporting care based on older adults’ health goals and needs. This study aimed to gain an understanding of volunteer experiences within the program and client and health care provider perspectives on the volunteer role.

**Methods:**

This study used a qualitative descriptive approach embedded in a pragmatic randomized controlled trial. Participants included Health TAPESTRY volunteers, health care providers, volunteer coordinator, and program clients, all connected to two primary care practice sites in a large urban setting in Ontario, Canada. Data collection included semi-structured focus groups and interviews with all participants, and the completion of a measure of attitudes toward older adults and self-efficacy for volunteers. Qualitative data were inductively coded and analyzed using a constant comparative approach. Quantitative data were summarized using descriptive statistics.

**Results:**

Overall, 30 volunteers and 64 other participants (clients, providers, volunteer coordinator) were included. Themes included: 1. Volunteer training: “An investment in volunteers”; 2. Intergenerational volunteer pairing: “The best of both worlds”; 3. Understanding the volunteer role and its scope: “Lay people involved in care”; 4. Volunteers as extensions of primary care teams: “Being the eyes where they live”; 5. The disconnect between volunteers and the clinical team: “Is something being done?”; 6. “Learning… all the time”: Impacts on volunteers; and 7. Clients’ acceptance of volunteers.

**Conclusions:**

This study showed that it is possible to integrate community volunteers into the primary care setting, adding human connections to deepen the primary care team’s understanding of their patients. Program implementation suggestions that emerged included: using role play in training, making volunteer role boundaries and specifications clear, and making efforts to connect volunteers and the primary care team they are supporting. This exploration of stakeholder voices has the potential to help improve volunteer program uptake and acceptability, as well as volunteer recruitment, retention, and training.

**Trial registration:**

For RCT: https://clinicaltrials.gov/ct2/show/NCT02283723, November 5, 2014.

## Background

Strong primary care systems are associated with improved population health; four main features describe system strength: first-contact access, long-term person-focused care, comprehensive care, and coordinated care [1–4]. Increasing the integration of community volunteers into the primary care setting has the potential to improve these key features. However, the use of volunteers in primary care is largely unexplored [5].

Volunteer programs in community and hospital settings have impacted a wide variety of patient health outcomes, including healthy behaviour changes [6–9]; decreased hospital admissions, stay length, or readmissions [10–12]; improved psychosocial outcomes [9, 13–15]; and increased self-efficacy for coping with cancer or managing diabetes [13, 16]. Volunteer roles in these programs have included: running physical activity programs [17, 18]; educating about topics like diet, diabetes management, cardiovascular health, and when to talk to a health care provider [10, 11, 19, 20]; home visiting [12, 14, 21, 22]; and health coaching or peer counselling [23–25]. In several instances, interventions delivered by volunteers were found to be as effective as those delivered by professionals [6, 15, 19, 26, 27].

Volunteering also has positive effects on the volunteers, with outcomes including better self-reported health and well-being, reduced depression, increased life satisfaction, fewer functional limitations, and lower mortality [28–30]. Volunteer training has been shown to foster protective factors, with arthritis peer support volunteers demonstrating better self-efficacy in arthritis self-management and decreased depressed mood post-training [31]. Volunteering also has an economic impact on the health care system: in studies of hospital and community-based volunteers the value of the benefits volunteers provided substantially exceeded the costs of running the volunteer programs [32–34]. Volunteer work is a crucial element that supports problem-solving worldwide, supporting individuals, communities, and organizations through unpaid, non-compulsory work [35]. However, despite the demonstrated benefits to clients, volunteers, and the system, reported in published literature, there seems to be little volunteer work occurring in Canadian primary care, and evaluation of those programs is even scarcer.

Health Teams Advancing Patient Experience: Strengthening Quality (Health TAPESTRY) is a community-based intervention rooted in primary care which aims to help people stay healthier for longer in the places where they live. It does this by sending trained volunteers equipped with eHealth technologies into older adults’ homes to serve as eyes and ears for interprofessional primary care teams [36]. These trained community volunteers are considered extensions of the primary care team. In a randomized controlled trial, clients receiving the Health TAPESTRY intervention had significantly fewer hospital admissions, spent less time sitting, and spent more time walking compared to controls [37].

The purpose of this study was twofold: first, to understand volunteers’ experiences of recruitment, training and program implementation and the impacts of volunteering on them; second, to explore client and health care provider perspectives on the volunteer role. The ultimate aim is to describe the structure of, and learnings from, this project to aid other primary care organizations interested in integrating volunteers into their setting.

## Methods

This study used a qualitative descriptive approach [38] embedded in a pragmatic randomized controlled trial (RCT) [36] to gain an understanding of the implementation of the volunteer program. The consolidated criteria for reporting qualitative research (COREQ) were used to report findings [39].

### Participants and setting

The study was based in an academic interprofessional primary care group practice with two clinic sites in a large urban area in Southern Ontario, Canada, serving over 36,000 patients. Participants were recruited from the pool of Health TAPESTRY volunteers who were at least 18 years old (or undergraduate students who were under the age of 18). Of the 143 community members who applied, 98 participated in screening and training to become Health TAPESTRY volunteers. Volunteers were categorized into two groups: novice (under 2 years of volunteer experience) and experienced (2 or more years of volunteer experience). All active Health TAPESTRY volunteers were invited to take part in this study through email. Other study participants included primary care team members and staff involved in delivering the Health TAPESTRY intervention, the volunteer coordinator, and clients receiving the intervention.

### Volunteers as a component of health TAPESTRY

Health TAPESTRY volunteers were initially recruited and screened through a multi-step process by Shalom Village, a local non-profit long-term care and supportive housing facility serving as the community volunteer partner [40]. Volunteers were trained through a multi-modal training program which included: a 2-h interactive, in-person training session; online learning through the Health TAPESTRY Virtual Learning Centre (VLC) including video modules and quizzes; and a paper-based manual. Content for training materials were developed through a literature review [41] and with input from volunteers and Shalom Village who provided continuous feedback and revisions. Examples of content for training included communications, program implementation and tools, health and safety, technology use, and privacy and confidentiality; for further detail see Oliver et al., 2018 [40].

The volunteers conducted home visits in mixed experience pairs (novice and experienced) to older adult clients (≥ 70 years of age), building relationships with them, and collecting information on client’s life and health goals, needs, and risks. They collected this information through questionnaires on clients’ life and health goals and needs on a secure online application (the Health TAPESTRY Application, or “TAP-App”) via tablet computers in the home. Visits lasted for approximately an hour, but volunteers could return if they did not complete all the questionnaires with the client. Overall, Health TAPESTRY volunteers conducted 657 visits to 360 clients. Completed questionnaires were collated into reports which were sent to the primary care team via the electronic medical record. Weekly interprofessional primary care “huddles” (a small team of 6 to 8 staff) triaged reports, coordinated further care, and planned next steps. A rich description of team functioning is described in another paper [42].

### Data collection and measures

To explore volunteer and provider perspectives, focus groups and interviews were conducted using a semi-structured interview guide informed by the core constructs of Normalization Process Theory [43] which underpinned the related qualitative evaluation of interprofessional team function embedded in the RCT. Questions were slightly adapted for each audience (see Table 1). Focus groups were held separately with experienced volunteers and novice volunteers (in a university building in the community) and members of the interprofessional huddle team (in their workplace) by paired facilitators; no one else was present. Volunteers were asked to complete an online Google Forms survey prior to the focus group, which included questions that required more direct or straightforward responses (e.g., “What is the goal or purpose of Health TAPESTRY?”). One-on-one interviews conducted by research staff were held with clients, the volunteer coordinator, and health care providers beyond the huddle team in person or over the phone. Participants were informed that the goal was to get their perspectives on the program and their experience in it. They received a $25 CAD gift card as a token of appreciation. Field notes were made by research staff after interviews and focus groups. Interviews and focus groups were audio-recorded and transcribed.

**Table 1:** Volunteer Focus Group Guide

Area	Question
Overall Understanding of Health TAPESTRY	What do you think is the goal or purpose of the Health TAPESTRY program?
	What do you think are the essential elements or parts of Health TAPESTRY?
	What do you think is the value or benefits of Health TAPESTRY for clients?
	Thinking of the Health TAPESTRY intervention overall, what are the things that are working well so far with Health TAPESTRY?
	Thinking about your experience in Health TAPESTRY so far:
	What do you value most deeply about your role as a Health TAPESTRY volunteer?
	Can you describe a time when you felt most fulfilled to be part of the Health TAPESTRY program?
	When did you felt most frustrated as a volunteer in the Health TAPESTRY program?
	How did you find the experience of working with a volunteer partner?
	What do you think are the things that are NOT working well so far?
	Do you think there are any drawbacks, threats or risks in using Health TAPESTRY, and if so, what are they?
	Would you recommend others to volunteer in Health TAPESTRY? Why or why not? Who do you think would be interested to volunteer?
Normalization of HEALTH Tapestry in Volunteer Practice	How do you understand your role (e.g. tasks and responsibilities) in relation to the Health TAPESTRY program?
	How do you understand your role in relation to others involved in Health TAPESTRY such as the health care team and clients?
	What, if anything, was done to make you feel that in your role as a volunteer, you were valued as an integral component of the Health TAPESTRY program?
	Do you feel that you had opportunities to give feedback and make improvements to the Health TAPESTRY program, and if yes, how?
Resources	How has the Health TAPESTRY program provided you with the knowledge, skills, and resources necessary to:
	Use the technological components of the program (VLC, TAP App, kindredPHR)?
	Navigate your clients to community resources?
	How well do you feel you were trained and prepared to be a Health TAPESTRY volunteer?
Impacts and Outcomes	How successful do you think the Health TAPESTRY program is in supporting older adults to identify and reach their life and health goals (goal-setting)?
	In what ways, if any, did Health TAPESTRY impact you?
	What opportunities came out of your participation in Health TAPESTRY that you would not otherwise have had?
Sustainability and Spread	From your experience of the TAPESTRY intervention so far, do you think Health TAPESTRY is sustainable (can keep going)?
	How do you see Health TAPESTRY being scaled up [spreading it wider]?
Closing	Do you have anything else to add?

Volunteer and provider sessions were held at two time points: Time 1 (months 5–7 of the rolling intervention) and Time 2 (intervention months 13–14); clients were invited to interviews at the end of their 6-month intervention period. Focus groups were 1–2 h; interviews averaged 40 min in length. Data collection continued until data saturation was reached (i.e., no new themes emerged). Interviewers and focus group facilitators included multiple research team members trained in qualitative research (MB, LC, NF, JG, DJ, LL, FP, and RV; 7 of whom were cisgender female, 1 genderqueer). Researchers may have been known to some volunteers through volunteer training, and to providers or clients through other studies.

Several surveys were also administered on paper at baseline as a way to describe the volunteer sample. Demographic information, including age, gender, level of education, country of birth, ethnicity, and marital status was collected. A measure of attitudes toward older adults (14 items, each rated on a 5-point Likert scale) [44] was also completed. The overall score ranged from 14 to 70 with higher scores indicating more positive attitudes toward older adults. Internal consistency was calculated and deemed satisfactory (α = 0.70).

Volunteers also completed a 3-item survey measuring their level of self-efficacy performing program tasks including: communicating with clients and asking about their current health, doing the different tasks and activities needed to manage or organize client visits on the TAP-App, and handling unexpected client issues or recognizing potential issues that providers should know about immediately. This survey was designed for this study and based on recommendations [45]. Items were scored from 0 to 100, with higher scores representing higher self-efficacy. Volunteers completed it approximately monthly on the TAP-App.

### Data analysis

Qualitative data were analyzed using the constant comparative approach [46]. RV and LC developed an initial coding structure using the interview questions and intervention components (volunteers, technology, interprofessional teams, and system navigation), and coded the first few transcripts to test the coding structure and adapt where required. The remaining transcripts were then independently, inductively coded by four team members (LC, JG, FP, and RV) within the overall coding structure. They met during monthly half-day meetings during the coding period to discuss meanings and codes, ensure consistency, and resolve any discrepancies through team discussion. NVivo 10 was used to organize the data [47]. The full research team reviewed the coding structure on three occasions to reach consensus on final themes and sub-themes. Methods used to foster rigour and trustworthiness included the use of Lincoln and Guba’s (1985) validation criteria (credibility, transferability, dependability, and confirmability) [48]; the use of multiple coders bringing different perspectives and experiences to data analysis including qualitative research and primary care; and the use of participants’ own words for authenticity.

We did not incorporate Normalization Process Theory as part of our analytic approach because as the RCT progressed it became apparent that the volunteers developed a unique role more distinct and less integrated into primary care practice that originally expected.

For quantitative data, the UCLA Geriatrics Attitudes Scale and the first and last completion of the self-efficacy scale by each volunteer were scored. Descriptive statistics were calculated for these scales and volunteer demographics. Analyses were completed in SPSS 25.

## Findings

### The population and sample

Thirty volunteers participated in this study, with 16 participating in focus groups at Time 1 (8 experienced, 8 novice) and 20 at Time 2 (10 experienced, 10 novice); 6 volunteers participated at both time points. Study volunteers ranged in age from 19 to 67, with most aged 25 and under and the remaining aged 60 and over. Most were female. More were students than other employment categories. Most of the retired volunteers (8 of 11; 72.7%) were retired health care professionals. Other participants included 31 health care providers and staff; 32 clients; and the volunteer coordinator. No participants dropped out as the requirement was just a single interview or focus group. See Table 2 for a description of all study participants.

**Table 2:** Participants in Focus Groups or Interviews

Participants	n (%)
Volunteers	30 (31.3)
Gender	
Female	20 (66.7)
Male	10 (33.3)
Age (years)	
25 or younger	17
60 or older	12
*missing age*	*1*
Category	
novice	16 (53.3)
experienced	14 (46.7)
Employment status	
student	15 (50.0)
retired	11 (36.7)
working or seeking employment	2 (6.7)
*missing employment status*	*2 (6.7)*
Original Recruitment Source	
university student centre	8 (26.7)
local/campus newspaper	8 (26.7)
word-of-mouth	6 (20.0)
poster/flyer	4 (13.3)
other	3 (10.0)
*missing recruitment source*	*1 (3.3)*
Clients	32 (33.3)
Age Category	
70–79 years	19 (59.4)
80 years +	13 (40.6)
Gender	
Female	16 (50.0)
Male	16 (50.0)
Health Care Providers and Staff	33 (34.4)
Type	
Family physician/medical resident	11 (33.3)
Allied health (dietitian, occupational therapist, physiotherapist, pharmacist, system navigator, psychologist)	11 (33.3)
Nurse (nurse practitioner, registered practical nurse)	7 (21.2)
Administrative support	2 (6.1)
Health care managers	2 (6.1)
Volunteer Coordinator	1 (1.0)
**Total**	**96 (100)**

Volunteers’ attitudes toward older adults scores ranged from 34 to 66 with a mean of 53.28 (SD = 7.93). Mean self-efficacy scores were high initially, and decreased slightly, with a larger change for experienced volunteers than for novice. See Table 3 for self-efficacy scores by volunteer.

**Table 3:** Self-Efficacy Scores

Volunteer & Category	Baseline Mean (SD)	Final Mean (SD)	Change in Mean	Times scale completed	Number of home visits
Vol-1 (E)	100.0 (0.0)	83.3 (11.5)	−16.7	11	56
Vol-3 (E)	93.3 (11.5)	90.0 (10.0)	−3.3	25	26
Vol-4 (E)	90.0 (10.0)	83.3 (20.8)	−6.7	10	18
Vol-8 (E)	90.0 (0.0)	83.3 (5.8)	−6.7	8	15
Vol-9 (E)	93.3 (11.5)	76.7 (15.3)	−16.6	24	72
Vol-10 (E)	100.0 (0.0)	90.0 (0.0)	−10.0	7	34
Vol-17 (E)	100.0 (0.0)	96.7 (5.8)	−3.3	9	24
Vol-18 (E)	100.0 (0.0)	100.0 (0.0)	0.0	12	34
Vol-19 (E)	96.7 (5.8)	Missing	N/A	1	37
Vol-20 (E)	83.3 (11.5)	80.0 (10.0)	−3.3	4	11
Vol-21 (E)	86.7 (5.8)	63.3 (5.8)	−23.4	11	16
Vol-22 (E)	90.0 (17.3)	76.7 (25.2)	−13.3	15	49
**E Total**	**93.6 (5.8)**	**83.9 (10.2)**	**−9.7 (7.3)**	**11.4 (7.1)**	**32.7 (18.5)**
Vol-5 (N)	90.0 (0.0)	83.3 (5.8)	−6.7	6	17
Vol-6 (N)	100.0 (0.0)	90.0 (0.0)	−10.0	4	28
Vol-7 (N)	96.7 (5.8)	80.0 (10.0)	−16.7	4	22
Vol-12 (N)	100.0 (0.0)	86.7 (5.8)	−13.3	4	19
Vol-13 (N)	80.0 (0.0)	86.7 (5.8)	6.7	4	6
Vol-14 (N)	86.7 (5.8)	90.0 (0.0)	3.3	2	6
Vol-16 (N)	90.0 (0.0)	80.0 (10.0)	−10.0	7	17
Vol-23 (N)	66.7 (5.8)	Missing	N/A	1	10
Vol-25 (N)	80.0 (10.0)	Missing	N/A	1	10
Vol-26 (N)	76.7 (5.8)	73.3 (5.8)	−3.4	4	11
Vol-27 (N)	56.7 (30.6)	66.7 (15.3)	10.0	3	17
**N Total**	**84.0 (13.7)**	**81.9 (7.8)**	**−2.1 (9.3)**	**3.6 (1.9)**	**14.8 (6.9)**
**TOTAL**	**89.0 (11.2)**	**83.0 (9.0)**	**−6.0 (8.4)**	**7.7 (6.5)**	**24.1 (16.6)**

*E* Experienced, *N* Novice, *SD* Standard Deviation; Scores range from 0 to 100, with higher scores representing higher self-efficacy

### Themes

Qualitative data from both time points are combined in the themes below, which are the key themes relating to the volunteer program across respondents. Sources are identified in the quotations as follows: volunteers as Vol-1, Vol-2, etc.; clients as C-01, C-02, etc.; huddle team members as Hud-1, Hud-2, etc.; and primary care team members beyond the huddle team as PC-1, PC-2, etc. No direct quotes from the volunteer coordinator are included to protect confidentiality, though responses were largely consistent with volunteers’ responses.

### Volunteer training: “An investment in volunteers”

Most volunteers found at least some of the training to be helpful, in particular the role play with simulated patients and technologies, food and social aspects, and ongoing “Lunch and Learn” sessions.

*“I personally enjoyed doing the simulated patients and creative things like that I found to be beneficial. They left the most lasting impact and I could actually remember everything that happened in those training sessions.” (Vol-6, Novice)*

Volunteers also identified some training gaps such as knowledge of local community resources. Several volunteers also described the difficulty of dealing with particularly sensitive or emotional situations, not feeling they fully understood the role volunteers could or should play in these scenarios, and not feeling fully prepared to deal with unexpected situations.

*“I’ve had a few interviews where we have people break down and cry and they’re grieving over a lost loved one, and it’s really hard, you know. I mean I’m not a social worker. I don’t pretend to be, and I don’t have the skills but what can you do, right.” (Vol-10, Experienced)*

Overall, they felt the training program was very robust compared to other volunteer positions.

*“It’s like an investment in the volunteers…” (Vol-12, Novice)*

However, the initial long gap of time between training and seeing clients was a challenge.

*“I feel bad because I feel like I’m the best trained volunteer in the history of the world, but now I have forgotten it all — well not really forgotten, but there was a long time frame.” (Vol-8, Novice)*

### Intergenerational volunteer pairing: “The best of both worlds”

Generally, volunteers appreciated being paired to share the work and feel safer and more comfortable in clients’ homes.

*“…Sometimes we are going into complete stranger’s homes right. We don’t know who these people are, so just having a partner there with you is very comforting." (Vol-16, Novice)*

Volunteers often described the intergenerational volunteer pairing as one of the strongest points of the program. It exposed volunteers to people in age groups other than their own. Older volunteers were generally identified as relating better to clients and having more depth of experience.

*“I think they often are able to relate better to the clients. I’ve had experiences where my partner has said something like, ‘Oh, I have like a daughter who’s going through a similar situation or, yeah, I get those aches and pains too’; Just kind of providing that empathy and forming that relationship better than I could do as a student.” (Vol-6, Novice)*

Younger volunteers were understood as having more technology knowledge and motivation to learn and experience.

*“A number of the partners I have been with don’t really know how to use an iPad or aren’t really … tech-savvy or comfortable with it. So in that way you get the best of both worlds.”* (*Vol-5, Novice)*

However, many volunteers commented that they tended to default to certain roles – younger volunteers entering information into the tablet, older volunteers taking the lead on facilitating conversation. Some novice volunteers expressed frustration over this.

*“It makes sense I think because our generation is more tech-savvy and it’s easier for us to immediately know how to navigate an iPad. But at the same time, it’s slightly frustrating because I wanted the opportunity to actually practice communication skills and speaking with a client.” (Vol-13, Novice)*

Pairing volunteers also came with some unexpected generational challenges, such as communication between the two groups.

*“…Sometimes it gets a bit difficult because sometimes some of them prefer phone calls and not emailing or texting which is faster for students, if you’re on the go, you’re like, can’t we text them?” (Vol-16, Novice)*

### Understanding the volunteer role and its scope: “Lay people involved in care”

Volunteers saw themselves as non-expert, flexible, committed community members on the front line of the project, supporting the health of their community.

*“… Just because it has to do with health doesn’t necessarily mean that it has to be health care professionals. If you can think of innovative ways for lay people to be involved in the care, as a community, to make people’s lives better and to have a share in community, I think that’s just really one of the highlights of [Health] TAPESTRY.” (Vol-1, Experienced)*

Volunteers understood their responsibilities to include keeping within boundaries, working confidentially, and basing interactions on client comfort.

*“There’s a responsibility of confidentiality obviously and presenting yourself in a professional manner and making them feel comfortable.” (Vol-4, Experienced)*

Yet at times they struggled to understand their role, particularly the scope of volunteer practice and amount of advice they could give.

*“…Sometimes I’m just really wanting to say something that I know would be helpful and I know I’m not sort of supposed to interact that way. Sometimes I’m just not exactly sure how much input we can give personally.” (Vol-1, Experienced)*

The main activities that volunteers saw as part of their role were: collecting information for the primary care team, listening to clients, helping clients think critically, and facilitating goal setting.

*“We’re not there to assist them in providing any sort of advice, but rather just to give them the ability to think about things critically in a way that they might have not thought about before, in that they can solve their own problems by thinking about what they need to address. . . and see what resources are available to them. And then we are almost like a mirror or something.” (Vol-14, Novice)*

Providers felt some functions were particularly suited to volunteers, such as transmitting information and some system navigation, while other issues had to be managed by the clinical team.

*“I see no reason why they couldn’t do some of the system navigation stuff at a first pass if there is an obvious need and an obvious resource and if they had training and information about local connections and resources, I think that would be very helpful. I think anything that would require any treatment has to come to the clinic, obviously.” (PC-1)*

However, multiple volunteers described feeling they had limited knowledge or ability to navigate clients to community resources, though some reported being able to provide at least some information.

*“Different volunteers know different things; like I personally am not from [City], so I can’t tell someone, ‘Oh did you know there’s a gym just down the road.’”* (*Vol-6, Novice)*

### Volunteers as extensions of primary care teams: “Be[ing] the eyes where they live”

Home visits allowed volunteers the space to see things and ask questions that the health care team may not have the access or time to explore.

*“I think that one of the values to them is that we get to be the eyes in where they live and see things that the health care team may not ever see. And obviously we are asking questions too that the health care team doesn’t always have time to explore in the normal course of their work with that patient.” (Vol-9, Experienced)*

Volunteers recognized that they were not bound by the time constraints of clinical health care, enabling them to collect rich information during the approximately 1-h home visits. On average, volunteers visited each client 3–4 times, including a scheduled 3-month follow-up visit.

*“Some clients would say that, I really appreciate the fact that someone actually came into my home and listened to what I had to say, without interrupting, without any time constraint; like you know, the doctor can only stay with me for 10 minutes or 15 minutes.” (Vol-15, Novice)*

Providers valued this benefit of volunteers, and for the most part saw volunteer home visits as extensions of the work they do.

*“The role that they are doing currently of going into the home and spending relaxed time with the person and covering all these assessment areas, I think itself is enormous.” (PC-4)*

### The disconnect between volunteers and the clinical team: “Is something being done?”

Volunteers described feeling removed from the health care team. While some understood how reports were sent to clinical staff, many were unsure what happened after they collected information or the impact on clients.

*“I’m submitting all this information, now where is it? It just feels like it’s just – where is it going? That was like the biggest question that I had. Who’s seeing this? Is something actually being done with this?” (Vol-12, Novice)*

Providers also experienced a disconnection between themselves and volunteers. They largely described having no direct connections to volunteers at all.

*“Direct exposure, none. So all of my understanding has been second-hand reports and anecdotes and stories and...” (PC-5)*

This lack of connection made it difficult for providers to understand what was happening on home visits and how they could use volunteers to the fullest extent.

*“We haven’t heard from the volunteers, so we really don’t know how all the interaction is happening with the patient… We’re not really getting a lot, and we probably haven’t thought outside the box a lot of how to use these volunteers in the loop around care. And we don’t know them, so it’s very hard to ask a stranger.” (Hud-8)*

To further add to the provider-volunteer disconnect, at times providers were uncertain how to interpret report, questioning if they could trust the information.

*“It’s not a specialist’s report or an investigation or something else that’s coming into our lab but it’s a volunteer…I don’t know much of what they have been trained with and how they are performing these kinds of assessments… So I think the aspect of a volunteer who I don’t know what training they’ve had for this assessment, plays a role into my thought process.” (Hud-9)*

Volunteers did feel very connected to the volunteer coordinator though, finding her to be strong in communication, scheduling, and showing volunteers are valued.

*“Even during the first Lunch & Learn, [Volunteer Coordinator] greeted me by name without even hesitating. I was amazed by that. I think that’s really, really important in terms of making sure volunteers feel valued and just making sure they are motivated to continue doing a good job.” (Vol-13, Novice)*

They also described support from the overall program team, particularly appreciating the opportunities to give and receive feedback, and the flexibility of the program to follow-up.

*“I like how there was lots of opportunity for feedback in terms of always asking the volunteers what was working, because we’re their front line in delivering those questionnaires. I know that people had concerns about the goals questionnaire and it was nice to see that the research team was being receptive of that feedback.” (Vol-14, Novice)*

However, volunteers described some logistics issues, including communication between volunteers, negotiating availability between volunteers with differing schedules, and transportation to client homes.

*“If [volunteer coordinator] asks two people if they are available on a day and time and one replies all and one doesn’t, the other one doesn’t know, okay, was she up for it, is it on, is it confirmed? And then you sit back and wait and you put a question mark.” (Vol-8, Experienced)*

### “Learning… all the time”: impacts on volunteers

Volunteering was a learning experience overall, with volunteers learning from their work, their partners, and their clients. Novice volunteers gained experiences that could be used in future careers or put on a résumé. Many volunteers described improvements to communication and active listening skills.

*“…If I’m not actively answering the questions I am able to absorb what they are saying and understand what they are saying. And that for me has been a really important learning skill because you’re talking about improving communication as well, but it’s just about as much as listening as it is about talking.” (Vol-12, Novice)*

Volunteers also felt that gaining exposure to older adults was a program benefit. Many described changing their views of older adults and awareness of the issues facing this population, including isolation, falls, and the spectrum of health presentations.

*“It helped me understand what 70-year-olds are all about because, you know, it sounds dumb but I have only ever known two 70-plus-year-olds; my grandma and my papa. And so it’s helpful to know more older adults and realize that they’re people like you and they have their own goals and just breaking pre-conceived notions about them.” (Vol-14, Novice)*

Volunteers felt that volunteering with Health TAPESTRY was purposeful, of value, and contributed to research and society, helping them feel pride in their contribution. Volunteers also felt excited to be part of something new in healthcare.

*“Being able to have the privilege to have the time to help them has had a very positive impact on me. I have done all types of different volunteer work, but when it’s one-on-one personal, it just kind of makes you feel proud; and it makes me feel proud of the community that offers it.” (Vol-18, Experienced)*

Volunteers were able to apply things they had learned previously, and the experience built value or added motivation to volunteers’ existing work, volunteering, and schooling.

*“I’m going into medicine next year and it kind of made me realize that there’s so many opportunities within medicine to develop new ideas and new ways of providing care.” (Vol-14, Novice)*

The understanding of healthy aging stemming from TAP-App questionnaires (on topics such as nutrition, physical activity, and mobility) was transferable to volunteers and their families, particularly with older volunteers.

*“I too am looking to this particular age group and am not far removed from that; I too have all along, every time I ask the questions I reflect back on my own experience too, and I think like where do I fit in this and what can I learn. And, so I find that I am learning as well all the time, which I love.” (Vol-3, Experienced)*

### Clients’ acceptance of volunteers

Volunteers were generally very well accepted by clients. Many clients felt satisfied with the volunteer home visits, finding them convenient. They described volunteers as friendly, pleasant, easy to talk to, respectful, and clear about describing the purpose of the program. Many expressed that having these visitors was a highlight of the program.

*“I sort of felt, I don’t know if it’s privileged, but wow, I’m getting these people coming here to sit with me and visit with me. And so I just felt it was a different experience.” (C-03)*

Only a few clients identified concerns, which were primarily about the skill and confidence of some volunteers.

*“I always felt that I was training them rather than that they were sort of training me, if that’s the right way of putting it*.” *(C-48)*

## Discussion

The aim of this study was to understand volunteers’ experiences in contributing to the care of older adults by extending the primary care team through home visits, and explore client and provider perspectives on this role. Collectively, our findings suggest that the volunteer program was well received. Home visits by volunteers allowed the space to spend more time with clients than clinical practice generally allows. Volunteers found meaning in their service to clients and felt valued through training, volunteer coordinator connections, and the ability to provide feedback. They felt that volunteering benefitted them in regards to communication skills, career experiences, exposure to different age groups, and reflections on their own health. Clients liked the volunteers and enjoyed the visits. Health care providers largely felt that there could be a role for volunteers in supporting primary care practice.

One challenge that emerged was the disconnected relationship between volunteers and providers. Achieving better connections between providers and volunteers is an important goal for future programs, whether that occurs through human connection, technology, or a combination. The College of Family Physicians of Canada describes the importance of health care team collaboration in supporting integrated, person-centred, coordinated care; their key competences for collaborative practice are: working effectively in a team-based model, cultivating and maintaining positive work environments, and recognizing and facilitating care transitions [49]. These skills can and should be extended to connected community volunteers who are supporting the primary care team.

There were also challenges around volunteer role clarity and specification. Roles for volunteers within primary care are largely not well fleshed-out. A report by Gilburt et al., describes the main primary care volunteer roles as: enabling general practice (e.g., driving, follow-up calls), sharing clinical space, social prescribing, and re-imagining a more community-set general practice [5]. Furthermore, a scoping review of system navigation in primary care showed that community workers or lay volunteers supported making clinic-community linkages [50]. Similarly, Health TAPESTRY volunteers did engage in some basic social prescribing (i.e., linkages to community health and social services), while also connecting with clients and collecting information, yet beyond that they sometimes struggled to define the scope of their practice. Interestingly, volunteers’ self-efficacy in role tasks dropped slightly from the beginning to end, with a larger drop for experienced volunteers, potentially indicating that they may have initially over-estimated their confidence, yet later realized what the role actually entailed. As the volunteer role is intended to complement paid health care providers, it is important to distinguish the boundaries of the volunteer role.

In terms of other aspects of the Health TAPESTRY volunteer program, from the volunteer perspective, the training was successful. Volunteers found that the most useful aspects were the role play through client simulations, social aspects, and ongoing learning. However, volunteers wished they could have more training on community resources and how volunteers could deal with sensitive situations. The initial time gap between training and seeing clients was also an issue. A study by Hainsworth et al. echoed the importance of these findings of the importance of socialization during training, as well as similar issues with a gap between training and volunteering [31]. Their delayed volunteers felt less motivated and more apprehensive to do the work, using terms like “let down” or “ignored”. These findings underscore the importance of closing the time gap between training and action.

In a study by Claxton-Oldfield et al. with hospice palliative care volunteers, researchers found that other common problems volunteers faced were: feeling underutilized, feeling undervalued by some medical staff, and not being able to do more to help patients [51]. Volunteers in our study also often wished they could do more. As many retired volunteers were health care providers and many students were in health fields, it may have been difficult not to be able to act on their full background and experience in this community volunteer role. Volunteers in our study had little connection to medical staff, yet felt valued by program staff responsible for their training and coordination. Generally, they felt well utilized. Chevrier et al., also working with hospice volunteers, found that aspects that gave volunteers the most satisfaction included: feeling like a team member, receiving feedback from the staff, feeling valuable, and having expectations match the position [52]. The team in our study, particularly the volunteer coordinator, made volunteers feel valued and volunteers appreciated the feedback that was both given and invited. However, the disconnection between volunteers and the primary care providers may have led to volunteers not feeling entirely like team members.

One unique aspect identified as favourable in Health TAPESTRY was the pairing between younger and older volunteers. Though the literature describes intergenerational volunteering programs, they generally describe older volunteers working with children, not paired volunteers of different age groups [53, 54]. These studies show benefits such as decreasing stress, perceived health benefits, and increasing sense of purpose and usefulness. Some of these benefits may have to do with the general benefits of volunteering on volunteers [29], yet our findings also show that working intergenerationally can have value, such as the meaning volunteers derived from being exposed to and learning from volunteers in different age groups than themselves.

Although we did not apply Normalization Process Theory in our analysis, we recognize that several of the themes which emerged are represented within NPT domains. These NPT domains include: Sense-Making, which can be seen in the volunteer training theme (what they are doing to understand their tasks and responsibilities); Cognitive Participation, which can be understood in both the intergenerational pairing and disconnect themes (as participants organize or reorganize to collectively contribute to the work); Coherence, which can be seen in understanding the volunteer role (making sense of the work); and Collective Action, which can be seen in the theme about volunteers as extensions of the primary care team (allocating the labour around operationalized practices).

### Strengths and limitations

We acknowledge several limitations in the study. First, volunteers self-selected into this evaluation component; not all Health TAPESTRY volunteers were included. Furthermore, the majority of volunteers were female and almost all were either under 25 or over 60, which could impact transferability. Chevrier et al. did find age and gender differences in volunteer satisfaction, with younger volunteers and men being more satisfied with external rewards (e.g., support, community respect, being underutilized), and older volunteers and women more satisfied with internal rewards (e.g., feeling their work is valuable, feeling they are impacting clients) [52]. The academic interprofessional primary care team setting and focus on Health TAPESTRY within this setting may also limit transferability, yet we believe findings have the potential to be applied to other multi-component, primary care-based programs involving volunteers. An adaptation of Health TAPESTRY focused on individuals with uncontrolled diabetes and hypertension also included a volunteer program evaluation. In this study, a framework for a volunteer program evaluation was created and findings extended the scope of volunteers and projects which could assist in understanding transferability of findings and in applying it to other settings [55].

A major strength of the research design was the inclusion of multiple different stakeholder voices including the volunteers themselves, clients, health care providers, and the volunteer coordinator, fostering transferability. Findings from qualitative data on other aspects of Health TAPESTRY beyond the volunteer component, including client experiences and the functioning of the interprofessional primary care team, has been published elsewhere [42, 56].

## Conclusions

Primary care-based volunteer programs are not common, particularly in Canada. The Health TAPESTRY volunteer program helped to fill this gap by creating a novel volunteer role which extended the work of primary care teams within clients’ homes Learnings from this volunteer program evaluation can be used to develop and implement further volunteer roles integrated into primary care. We found that volunteers in this program truly were able to complement the roles of primary care providers without much overlap in their tasks, yet volunteers wished they had more of a connection to providers. Mixed experience level pairing, which resulted in intergenerational pairing, provided a unique benefit to the volunteers and the facilitation of the program; within these pairings the volunteers were able to develop their own practical skills and were better able to carry out their tasks. A well-developed volunteer training curriculum was a facilitator of the program, but volunteers wished they had a more clearly defined role. Overall, volunteers felt valued and that their work was meaningful, with the key barriers to a more successful program being largely logistical.

## Data Availability

All data generated and analyzed during the current study are not publicly available due to the confidential nature of participant transcript data, but are available from the corresponding author on reasonable request.

## Abbreviations

COREQ: Consolidated Criteria for Reporting Qualitative Research
Health TAPESTRY: Health Teams Advancing Patient Experience: Strengthening Quality
RCT: Randomized Controlled Trial
TAP-App: Health TAPESTRY Application
